# Gnaphalium in Sciatica*Read before the Central N. Y. Hom. Society, at Syracuse, March, 1882.

**Published:** 1882-04

**Authors:** S. Swan

**Affiliations:** New York


### GNAPHALIUM IN SCIATICA.*

S. Swan, M. D. New York.

Mr. K——, suffering from caries of the right femur, the result of an accident many years since, was attacked with a severe sciatic pain, commencing inside of the pelvis, and following the course of the sciatic nerve to the knee. The pain was intense and unbearable, and he begged for a hypodermic injection. On a careful study of the case and remedies, Gnaphalium showed this symptom in Allen, italicised, "*intense pain along the sciatic nerve.*" Here was a case calling for prompt action, and as my experience had shown me that the highest potencies acted quickest, a dose of the CMM was put in half-tumbler of water, and a tea-spoonful given every fifteen minutes; before the third dose, the pain had gone, and the patient was sleeping quietly; there was no return of the sciatica.

*Read before the Central N.Y. Hom. Society, at Syracuse, March, 1882.

